# Survival Associated With Sirolimus Plus Tacrolimus Maintenance Without Induction Therapy Compared With Standard Immunosuppression After Lung Transplant

**DOI:** 10.1001/jamanetworkopen.2019.10297

**Published:** 2019-08-28

**Authors:** Marniker Wijesinha, Jon Mark Hirshon, Michael Terrin, Laurence Magder, Clayton Brown, Kristen Stafford, Aldo Iacono

**Affiliations:** 1 Department of Epidemiology and Public Health, University of Maryland School of Medicine, Baltimore; 2 Department of Emergency Medicine, University of Maryland School of Medicine, Baltimore; 3 Department of Medicine, University of Maryland School of Medicine, Baltimore

## Abstract

**Importance:**

Median survival after lung transplant is less than 6 years. Standard maintenance therapy typically includes tacrolimus and an antimetabolite (mycophenolate mofetil or azathioprine). Replacing the antimetabolite with sirolimus after postoperative wound healing may improve long-term survival due to antifibrotic, antiproliferative, and antiaging effects of sirolimus.

**Objectives:**

To compare survival between patients receiving sirolimus plus tacrolimus vs mycophenolate mofetil plus tacrolimus (the most common maintenance therapy) and to identify the combination of induction and maintenance therapy associated with the highest survival.

**Design, Setting, and Participants:**

This cohort study of US recipients of lung transplants from January 1, 2003, through August 31, 2016, analyzed United Network for Organ Sharing (UNOS) data from January 1 through September 13, 2018. Because initiation of sirolimus therapy is usually delayed 3 to 12 months after lung transplant, primary analyses were based on patients alive and free of chronic rejection and malignant disease at 1 year in all groups, whereas sensitivity analyses used appropriate methods to include all patients from transplant time. Regression models adjusted for available potential confounders, including transplant center performance.

**Exposures:**

Cell cycle inhibitor maintenance therapies, including sirolimus (n = 219), mycophenolate mofetil (n = 5782), mycophenolate sodium (n = 408), azathioprine (n = 2556), and concurrent sirolimus plus mycophenolate mofetil (n = 54), were compared within a tacrolimus-based regimen. Combinations of each induction (basiliximab, daclizumab, antithymocyte globulin, alemtuzumab, or none) and maintenance (tacrolimus plus sirolimus, mycophenolate mofetil, or azathioprine) therapy were also compared.

**Main Outcomes and Measures:**

Survival was the primary outcome; chronic rejection incidence and subsequent mortality were secondary outcomes.

**Results:**

Among this population of 9019 patients (median age, 57 years [interquartile range {IQR}, 46-63 years]; 5194 men [57.6%]), sirolimus plus tacrolimus was associated with better survival than mycophenolate mofetil plus tacrolimus (median, 8.9 years [IQR, 4.4-12.7 years] vs 7.1 years [IQR, 3.6-12.1 years]; adjusted hazard ratio [aHR], 0.71; 95% CI, 0.56-0.89; *P* = .003). Chronic rejection incidence (aHR, 0.75; 95% CI, 0.61-0.92) and mortality after chronic rejection (aHR, 0.52; 95% CI, 0.31-0.81) were lower with sirolimus plus tacrolimus. Compared with mycophenolate mofetil plus tacrolimus, survival differences for sirolimus plus mycophenolate mofetil plus tacrolimus (aHR, 1.14; 95% CI, 0.79-1.65), mycophenolate sodium plus tacrolimus (aHR, 0.95; 95% CI, 0.77-1.17), and azathioprine plus tacrolimus (aHR, 0.93; 95% CI, 0.84-1.02) were not significant. The induction-maintenance combination with the highest survival was sirolimus plus tacrolimus without induction therapy (median survival, 10.7 years [IQR, 7.3-12.7 years]; aHR, 0.48; 95% CI, 0.31-0.76; *P* = .002) compared with mycophenolate mofetil plus tacrolimus with induction therapy (median survival, 7.4 years [IQR, 3.9-12.6 years]).

**Conclusions and Relevance:**

Sirolimus plus tacrolimus was associated with improved patient survival after lung transplant compared with mycophenolate mofetil plus tacrolimus; no antibody induction therapy with sirolimus plus tacrolimus was associated with maximal survival.

## Key Points

**Question:**

Which immunosuppressive strategy is associated with the highest survival in lung transplantation?

**Findings:**

In this cohort study of US recipients of lung transplants, sirolimus plus tacrolimus was associated with significantly better conditional survival from 1 year after transplant compared with mycophenolate mofetil plus tacrolimus (median survival, 8.9 vs 7.1 years after transplant). The highest conditional survival was observed in patients receiving sirolimus plus tacrolimus with no induction therapy (median survival, 10.7 years), which was significantly better than survival for those receiving mycophenolate mofetil plus tacrolimus with induction therapy (median survival, 7.4 years).

**Meaning:**

Sirolimus plus tacrolimus was associated with improved patient survival compared with mycophenolate mofetil plus tacrolimus, and using no induction therapy with sirolimus plus tacrolimus was associated with the highest survival.

## Teaser

This cohort study compares survival of US recipients of lung transplants receiving sirolimus plus tacrolimus vs standard maintenance therapy and evaluates which combination of induction and maintenance therapy is associated with the highest survival.

## Introduction

Lung transplant is life prolonging for patients with end-stage lung disease, but high mortality limits median posttransplant survival to less than 6 years. Patients receive lifelong immunosuppressive regimens to prevent eventual lung function loss owing to chronic lung allograft dysfunction resulting from chronic rejection, which usually manifests as bronchiolitis obliterans syndrome or restrictive allograft syndrome.^1,2^ Unfortunately, immunosuppressive regimens are not completely effective in preventing chronic rejection, and many patients die of immunosuppression-related consequences, including infections and malignant disease. No immunosuppressive drug currently has US Food and Drug Administration approval for patients who have received a lung transplant, so all drugs are used off-label.

Standard long-term immunosuppressive regimens combine a calcineurin inhibitor (tacrolimus or cyclosporine) with an antimetabolite (mycophenolate mofetil, mycophenolate sodium, or azathioprine), which serves as a cell cycle inhibitor. An alternative regimen replaces the antimetabolite with a mammalian target of rapamycin inhibitor (most commonly sirolimus) as the cell cycle inhibitor. This replacement usually occurs 3 to 12 months after transplant because mammalian target of rapamycin inhibitors can impair wound healing if administered immediately after lung transplant, leading to bronchial anastomotic dehiscence, which is frequently fatal.^3^

Potential benefits of sirolimus include multiple mechanisms for preventing chronic rejection^4,5,6,7^ and anticancer^8,9,10,11^ and antiaging^10,12^ effects. Sirolimus has been an effective rescue therapy in lung transplantation after chronic rejection^7^ or skin cancer^13^ occurred with other immunosuppressants. The limited extent of prior studies^6,14,15,16^ on prophylactic sirolimus use in lung transplantation has led to inconclusive findings, although results were favorable in most studies. A randomized open-label 3-year study^6^ comparing sirolimus (therapy initiated 3 months after transplant) with azathioprine, in which more than half the patients in an intention-to-treat population discontinued their assigned therapy, found no significant differences besides lower cytomegalovirus infection incidence with sirolimus. A single-center long-term cohort study^14^ found that sirolimus (therapy initiated 1 year after transplant) was significantly superior to mycophenolate mofetil in survival (67% vs 37% alive at 9 years), with lower chronic rejection and infection incidence. Another single-center study^15^ found that patients receiving sirolimus within 6 months after transplant had better lung function at 3 years. Finally, a recent single-center study^16^ reported favorable short-term and long-term survival and very low incidence of chronic rejection when sirolimus was initiated 1 month after transplant in patients with completely healed bronchial anastomoses. Because no large-scale studies, to our knowledge, have examined long-term survival associated with prophylactic sirolimus use in lung transplantation, the primary goal of this study was to compare long-term survival between patients receiving sirolimus and mycophenolate mofetil, the most popular cell cycle inhibitor, using national US lung transplant data.

## Methods

### Study Design and Population

This retrospective cohort study included US recipients of lung transplants from January 1, 2003, through August 31, 2016, in the United Network for Organ Sharing (UNOS) data set. The institutional review board of the University of Maryland, Baltimore, determined that this study did not constitute human subjects research, did not require institutional review, and was exempt from the need for informed consent. We followed the Strengthening the Reporting of Observational Studies in Epidemiology (STROBE) reporting guidelines for cohort studies. All study patients received the calcineurin inhibitor tacrolimus (Prograf) prophylactically and received no antibody induction therapy or antibody induction therapy with 1 of the following: basiliximab, daclizumab, alemtuzumab, or antithymocyte globulin. Cell cycle inhibitors studied included sirolimus (Rapamune), mycophenolate mofetil (Cellcept), mycophenolate sodium (Myfortic), azathioprine (Imuran), and the combination of sirolimus and mycophenolate mofetil. Patients who did not receive one of these drugs for prophylactic immunosuppression or who received a drug or combination not mentioned (besides corticosteroids) were excluded.

Patients were classified based on the cell cycle inhibitor they received prophylactically, which can generally be found in their first immunosuppression record in the data set, just after transplant. However, because sirolimus initiation is usually delayed for 3 to 12 months after transplant, most sirolimus-treated patients must be identified from a follow-up immunosuppression record in the data set (occasionally available at 3 or 6 months but usually available only at 1 year after transplant or at death for patients who died within the first year). Because sirolimus use in the first year is sometimes for rescue rather than prophylactic use, commonly after the onset of chronic rejection or malignant disease^7,13,17^ (which are both reported annually in the UNOS data set), patients experiencing chronic rejection or malignant disease in the first year would be more likely to receive sirolimus. To avoid this potential source of confounding, primary analyses were based on patients free of chronic rejection and malignant disease (and alive) at 1 year after transplant in all treatment groups. For all analyses, patients were retained in their prophylactic treatment group classifications regardless of any eventual treatment switches.

### Statistical Analysis

All analyses were performed using SAS, version 9.4 (SAS Institute Inc) or R, version 3.4.3 (R Project for Statistical Computing) from January 1 through September 13, 2018. A 2-sided *P* < .05 was considered statistically significant. Between the patients treated with sirolimus (sirolimus group) and mycophenolate mofetil (MMF group, ie, the largest group), continuous variables were compared using Wilcoxon rank sum tests, accommodating nonnormal distributions, and categorical variables were compared using χ^2^ tests. Variables included age, sex, race, body mass index, educational attainment, smoking history (>10 pack-years), diabetes, poor renal function (creatinine level ≥1.3 mg/dL [to convert to micromoles per liter, multiply by 88.4]), lung disease type, medical condition, prior transplant, donor-recipient HLA matching, donor-recipient cytomegalovirus status, donor-to-recipient predicted total lung capacity ratio, donor age, donor sex, donor smoking (>20 pack-years), transplant type, administration of high-dose corticosteroids at transplant, Lung Allocation Score (LAS), transplant era (2003 to 2005 pre-LAS, 2005 post-LAS to 2010, or 2011 to 2016), and induction drug.

For survival comparisons between groups, we explored multiple approaches to avoid immortal time bias resulting from delayed initiation of sirolimus therapy. The primary approach involved Cox proportional hazards regression (generating adjusted hazard ratios [aHRs] and 95% CIs) and Kaplan-Meier survival analyses starting at a landmark time^18,19^ of 12 months after transplant such that all patients included in the analysis were alive at the landmark time. A landmark time of 3 months was also explored to confirm similarity of results. These 2 landmark times correspond to sirolimus therapy initiation occurring hypothetically as early as possible (3 months after transplant) or as late as possible within the first year (12 months after transplant) because exact sirolimus therapy initiation times are unavailable. In context of a 3-month sirolimus therapy initiation time, the possibility of rescue sirolimus use is less likely, so all patients receiving sirolimus within the first year were counted as prophylactic users. However, in context of a 12-month sirolimus therapy initiation time, if a patient had chronic rejection or malignant disease recorded within the first year, this outcome would have occurred before sirolimus therapy initiation and is likely the reason for initiation; therefore, patients experiencing chronic rejection or malignant disease in the first year were excluded from all treatment groups to ensure comparable groups and avoid confounding by indication. The second analytical approach used a time-dependent covariate for sirolimus in a Cox proportional hazards regression model, with either 12 or 3 months considered as the initiation time for sirolimus therapy. The third approach used multiple imputation to identify, among patients dying within 12 months after transplant, those who were likely planned to receive sirolimus (but died before they could receive it). Based on demographic, clinical, or transplant characteristics associated with patients in the sirolimus group, patients dying within the first year who had similar characteristics were identified via multiple imputation and added to the sirolimus group, and survival analyses were performed starting at transplant time.

In regression analyses, all aforementioned variables except LAS were adjusted for, because LAS is largely based on several of these variables and some patients in our study underwent transplant before LAS introduction. Regression models included a random effect representing transplant center to address confounding by center. Multiple imputation was used to enable regression analyses to include patients with missing covariate data. Twenty imputed data sets were generated using the fully conditional specification method,^20^ and mean regression coefficients were calculated from results based on these 20 imputed data sets.

We examined frequencies of deaths due to each common cause (rejection/pulmonary, infection, malignant disease, other organ failure, or other/unknown) in the sirolimus and MMF groups based on cumulative incidence functions. For the 3 most common mortality-causing events (chronic rejection, infection, and malignant disease), semicompeting risks Cox proportional hazards regression analyses compared event risks and postevent death risks between drugs. The semicompeting risks framework, intended to address possible informative censoring of nonfatal events by death due to other causes, is explained elsewhere by Alvares et al^21^ and Haneuse and Lee,^22^ authors of the *SemiCompRisks R* package. A reference manual is available at https://cran.r-project.org, and these methods have been previously applied by Jazić et al.^23^

Finally, to assess whether the survival comparisons between sirolimus and antimetabolites differ according to the induction therapy used (if any), we examined survival associated with each possible combination of induction (alemtuzumab, antithymocyte globulin, basiliximab, daclizumab, or no induction) and maintenance (tacrolimus plus sirolimus, mycophenolate mofetil, or azathioprine) therapies. This analysis enabled us to determine which induction-maintenance combination was associated with the highest survival.

## Results

### Comparison of Patient, Donor, and Transplant Characteristics Between Groups

The study population for the main analysis consisted of 9019 recipients of lung transplants with a median age of 57 years (interquartile range [IQR], 46-63 years), including 5194 men (57.6%) and 3825 women (42.4%). Table 1 compares major characteristics between the sirolimus and MMF groups. Most characteristics were similar between groups; any differences were adjusted for in regression analyses.

**Table 1.** Patient Characteristics at Time of Transplant

Characteristic	Study Group^a^		*P* Value^b^
	Sirolimus (n = 219)	Mycophenolate Mofetil (n = 5782)	
Age, median (IQR), y	58 (48-63)	58 (46-64)	.53
Sex			
Male	128 (58.4)	3326 (57.5)	.79
Female	91 (41.6)	2456 (42.5)	
Race/ethnicity			
Black	27 (12.3)	456 (7.9)	.05
White	178 (81.3)	4788 (82.8)	
Hispanic	9 (4.1)	407 (7.0)	
Other	5 (2.3)	131 (2.3)	
BMI category			
Underweight	38 (17.4)	1115 (19.5)	.34
Normal	62 (28.4)	1764 (30.9)	
Overweight	76 (34.9)	1982 (34.7)	
Obese	42 (19.3)	859 (15.0)	
Lung disease (primary)			
Cystic fibrosis	31 (14.2)	881 (15.2)	.70
Pulmonary fibrosis	84 (38.4)	2251 (38.9)	
COPD	63 (28.8)	1535 (26.5)	
α1-Antitrypsin deficiency	3 (1.4)	175 (3.0)	
Pulmonary hypertension	6 (2.7)	181 (3.1)	
Sarcoidosis	9 (4.1)	163 (2.8)	
Other	23 (10.5)	596 (10.3)	
Prior transplant			
Yes	17 (7.8)	233 (4.0)	.007
No	202 (92.2)	5549 (96.0)	
LAS			
Median (IQR)	41 (35-48)	40 (35-50)	.97
Missing or NA (pre-LAS era)	70 (32.0)	581 (10.0)	
Transplant type			
Single	89 (40.6)	2056 (35.6)	.12
Double	130 (59.4)	3726 (64.4)	
Induction therapy			
Alemtuzumab	15 (6.8)	504 (8.7)	<.001
Equine ATG	4 (1.8)	323 (5.6)	
Rabbit ATG	2 (0.9)	266 (4.6)	
Basiliximab	71 (32.4)	1890 (32.7)	
Daclizumab	60 (27.4)	467 (8.1)	
No induction	67 (30.6)	2332 (40.3)	
HLA matching			
0-3 Matches	171 (96.6)	4927 (96.1)	.75
4-6 Matches	6 (3.4)	198 (3.9)	
Donor age, median (IQR), y	30 (20-44)	31 (21-45)	.48
Donor smoking (>20 pack-years)			
Yes	37 (16.9)	637 (11.1)	.008
No	182 (83.1)	5082 (88.9)	

Abbreviations: ATG, antithymocyte globulin; BMI, body mass index; COPD, chronic obstructive pulmonary disease; HLA, human leukocyte antigen; IQR, interquartile range; LAS, Lung Allocation Score; NA, not available.

^a^ Unless otherwise specified, data are expressed as number (percentage) of patients. Percentages have been rounded and may not total 100.

^b^ Calculated using χ^2^ tests for categorical variables and Wilcoxon rank sum tests for continuous variables.

### Comparison of Survival Between Sirolimus and MMF Groups

**Figure 1.**
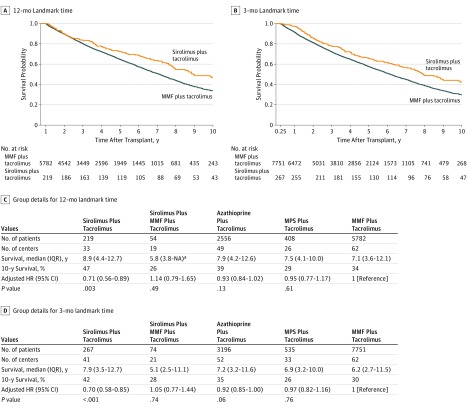
Survival for Sirolimus vs Mycophenolate Mofetil (MMF) and Other Standard Therapies HR indicates hazard ratio; IQR, interquartile range; MPS, mycophenolate sodium; and NA, not applicable. ^a^There is no upper value for this range because follow-up ended before the 75th percentile of survival time was reached.

In the 12-month landmark time analysis, the aHR for the sirolimus group vs MMF group was  0.71 (95% CI, 0.56-0.89; *P* = .003). Median survival was 8.9 years (IQR, 4.4-12.7 years) vs 7.1 years (IQR, 3.6-12.1 years) after transplant for the sirolimus vs MMF groups, respectively. In the 3-month landmark time analysis, the aHR was 0.70 (95% CI, 0.58-0.85; *P* < .001) and median survival was 7.9 years (IQR, 3.5-12.7 years) vs 6.2 (IQR, 2.7-11.5 years) for the sirolimus vs MMF groups. Figure 1 displays the corresponding Kaplan-Meier survival curves. All other analytical approaches also indicated better survival for the sirolimus than the MMF groups, as shown in eTable 1 and eFigure 1 in the Supplement. In addition, sirolimus was associated with the most favorable survival among all alternatives assessed (sirolimus plus mycophenolate mofetil, mycophenolate sodium, and azathioprine) based on all approaches. No other therapy had significantly different survival compared with mycophenolate mofetil (aHR for sirolimus plus mycophenolate mofetil plus tacrolimus, 1.14 [95% CI, 0.79-1.65]; aHR for mycophenolate sodium plus tacrolimus, 0.95 [95% CI, 0.77-1.17]).

### Comparison of Incidences and Mortality Due to Major Causes of Death Between Groups

**Figure 2.**
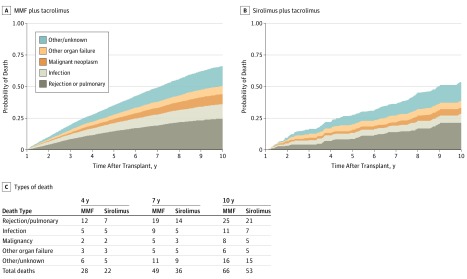
Probability of Death by Major Causes (Sirolimus vs Mycophenolate Mofetil [MMF])

For the sirolimus group and MMF group, Figure 2 displays frequencies of deaths from each major cause starting at 12 months after transplant. In semicompeting risks multivariable regression analyses, the sirolimus group had lower chronic rejection incidence than the MMF group (aHR, 0.75; 95% CI, 0.61-0.92; *P* = .005) and lower death risk after chronic rejection (aHR, 0.52; 95% CI, 0.31-0.81; *P* = .009). Incidence of infection was similar (aHR, 0.91; 95% CI, 0.55-1.36; *P* = .68), but death risk after infection was lower in the sirolimus group (aHR, 0.33; 95% CI, 0.21-0.53; *P* < .001). We were unable to detect a significant difference in malignant disease incidence (aHR, 0.71; 95% CI, 0.47-1.03; *P* = .09) or mortality risk after malignant disease (aHR, 0.70; 95% CI, 0.37-1.23; *P* = .26) in the sirolimus group.

### Assessment of Factors Influencing Survival Among Sirolimus-Treated Patients

Among the sirolimus group, 124 patients (66.7%) continued sirolimus therapy 1 year later (at the 2-year follow-up). Among patients alive at 2 years, survival was significantly better for those who continued sirolimus therapy at year 2 compared with those who discontinued (median survival, 10.5 years [IQR, 6.6 years to not reached] vs 7.8 years [IQR, 5.1-12.7 years]; *P* = .01) as shown in eFigure 2 in the Supplement (for the sirolimus group, follow-up ended before the 75th percentile of survival time was reached). A similar trend, but a smaller survival difference, was observed for those who continued vs discontinued mycophenolate mofetil therapy at year 2 in the MMF group (median survival, 8.1 years [IQR, 4.7-12.5 years] vs 7.2 years [IQR, 4.5-11.3 years]; *P* < .001). Patients who continued sirolimus therapy at year 2 had better survival than those who continued mycophenolate mofetil therapy at year 2, within each group (median survival, 10.5 years [IQR, 6.6 years to not reached] vs 8.1 years [IQR, 4.7-12.5 years]; *P* = .006).

Within sirolimus-treated patients, long-term survival did not significantly differ by the antimetabolite initially used before initiation of sirolimus therapy (mycophenolate mofetil [reference], azathioprine [HR, 0.72; 95% CI, 0.27-1.91], or mycophenolate sodium [HR, 1.12; 95% CI, 0.43-2.95]; *P* = .26). As shown in eTable 2 in the Supplement, the aHRs for use of each induction drug compared with no induction were all greater than 1.00, suggesting that survival was not better, and possibly worse, if any antibody induction therapy was given, among sirolimus-treated patients.

### Comparison of Survival Between Induction and Maintenance Therapy Combinations

Among combinations of induction and tacrolimus-based maintenance therapies, within patients alive at 1 year, sirolimus with no induction therapy had the most favorable survival (10.7 years [IQR, 7.3-12.7 years]; aHR, 0.41; 95% CI, 0.26-0.64; *P* < .001) compared with the most common combination, mycophenolate mofetil with no induction (6.8 years [IQR, 2.2-10.5 years]). Table 2 shows results for each induction-maintenance combination. Among the 67 patients who received sirolimus with no induction, 23 (34.3%) were administered high-dose corticosteroids at the time of transplant. High-dose corticosteroid administration was not associated with overall mortality (HR, 0.85; 95% CI, 0.24-3.02; *P* = .95); patients who received it had fewer deaths due to rejection but more deaths due to infection and other causes.

**Table 2.** Survival Comparisons Between Maintenance and Induction Therapy Combinations^a^

Rank	Maintenance Therapy^b^	Induction Therapy	aHR (95% CI) for Death	*P* Value^c^	No. of Patients	No. of Centers
1	Sirolimus	None	0.41 (0.26-0.64)	<.001	67	25
2	Sirolimus	Daclizumab	0.57 (0.38-0.88)	.01	60	8
3	Azathioprine	Equine ATG	0.75 (0.54-1.03)	.08	149	9
4	Mycophenolate mofetil	Daclizumab	0.79 (0.66-0.94)	.009	467	25
5	Mycophenolate mofetil	Alemtuzumab	0.79 (0.62-1.02)	.07	504	7
6	Mycophenolate mofetil	Equine ATG	0.80 (0.64-1.01)	.06	323	12
7	Azathioprine	Daclizumab	0.82 (0.66-1.02)	.08	269	15
8	Azathioprine	Basiliximab	0.82 (0.70-0.97)	.02	972	30
9	Sirolimus	Basiliximab	0.87 (0.60-1.27)	.48	71	18
10	Mycophenolate mofetil	Basiliximab	0.89 (0.78-1.01)	.07	1890	52
11	Azathioprine	None	0.90 (0.79-1.02)	.09	1136	42
12	Mycophenolate mofetil	None	1 [Reference]	NA	2332	58
13	Mycophenolate mofetil	Rabbit ATG	1.15 (0.90-1.46)	.27	266	26

Abbreviations: aHR, adjusted hazard ratio; ATG, antithymocyte globulin; NA, not applicable.

^a^ The following combinations had small numbers of patients and are not shown in the table: (1) azathioprine and rabbit ATG, (2) sirolimus and alemtuzumab, (3) azathioprine and alemtuzumab, (4) sirolimus and equine ATG, and (5) sirolimus and rabbit ATG.

^b^ Indicates cell cycle inhibitor within a tacrolimus-based regimen.

^c^ Calculated using Cox proportional hazards regression model with a Wald test.

**Figure 3.**
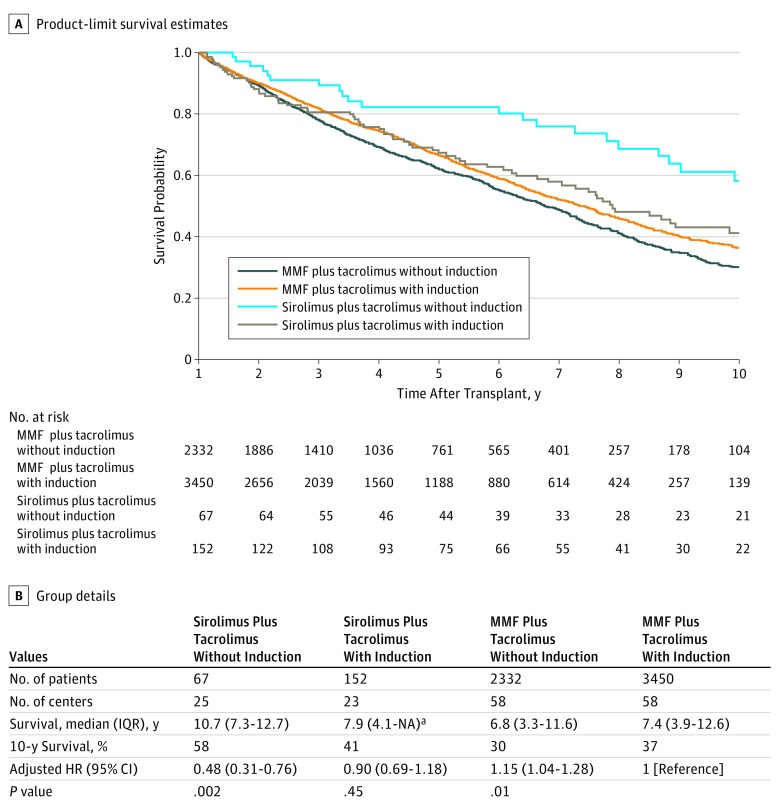
Survival for Sirolimus vs Mycophenolate Mofetil (MMF), With or Without Induction Therapy Types of induction therapy are alemtuzumab, antithymocyte globulin, basiliximab, and daclizumab. HR indicates hazard ratio; IQR, interquartile range; and NA, not applicable. ^a^There is no upper value for this range because follow-up ended before the 75th percentile of survival time was reached.

Figure 3 displays Kaplan-Meier survival curves (starting at 1 year) for the sirolimus and MMF groups, with or without any induction. Compared with mycophenolate mofetil with induction, sirolimus with no induction had significantly better survival (median survival, 10.7 [IQR, 7.3-12.7 years] vs 7.4 years [IQR, 3.9-12.6 years]; aHR, 0.48; 95% CI, 0.31-0.76; *P* = .002). These findings persisted in analyses starting at the time of transplant based on multiple imputation–derived identification of planned sirolimus treatment, as shown in eFigure 3 in the Supplement. A significant interaction occurred between the maintenance and induction variables (*P* for interaction = .002); results suggested that induction was detrimental among sirolimus-receiving patients (HR for induction, 1.81; 95% CI, 1.09-3.02) but slightly beneficial within mycophenolate mofetil–receiving patients (HR for induction, 0.86; 95% CI, 0.77-0.96). Starting from the time of transplant (among antimetabolite-receiving patients), induction therapy was not associated with improved survival to 1 year after transplant (ie, until sirolimus therapy might be initiated), as shown in eFigure 4 in the Supplement. In addition, survival to 1 year after transplant did not differ (*P* = .21) according to the antimetabolite initially used for maintenance therapy from transplant time: mycophenolate mofetil (reference), azathioprine (HR, 0.91; 95% CI, 0.78-1.05), and mycophenolate sodium (HR, 0.80; 95% CI, 0.58-1.11).

## Discussion

To our knowledge, this study provides the first report on long-term survival for prophylactic (or nearly prophylactic) sirolimus use based on national US lung transplant data. All analytical approaches indicated that sirolimus therapy was associated with better survival than mycophenolate mofetil and also with the most favorable survival among alternatives, including sirolimus plus mycophenolate mofetil, azathioprine, and mycophenolate sodium, within a tacrolimus-based regimen.

Previously, a randomized open-label trial by Bhorade et al^6^ compared sirolimus therapy initiated 3 months after transplant with azathioprine therapy and found no significant differences in 3-year survival, whereas a cohort study by Sacher et al^14^ found that sirolimus therapy initiated 1 year after transplant was associated with significantly better survival than mycophenolate mofetil, and the improvement was sustained for the follow-up period of 10 years. The different study durations may partly explain the discrepant findings because short-term mortality is relatively low and commonly unrelated to immunosuppressive efficacy or adverse effects, so immunosuppression-related survival differences may only manifest later. In fact, fairly consistent with the findings of the randomized study by Bhorade et al,^6^ which ended at 3 years after transplant, our results suggest that the survival benefit of sirolimus may only start to manifest approximately 2 to 3 years after transplant (considering that some patients only began sirolimus therapy at 1 year after transplant), although this benefit is considerable in the long term. Another factor differentiating the studies by Bhorade et al^6^ and Sacher et al^14^ is that nearly half the patients in the study by Bhorade et al^6^ discontinued sirolimus therapy within 1 year of initiation compared with only 21% in the latter study by Sacher et al.^14^ Sacher et al^14^ attributed this superior retention to delaying initiation of sirolimus therapy to 1 year after transplant; medications toxic to bone marrow, including valganciclovir hydrochloride and voriconazole, were discontinued by then, and sufficient kidney recovery time had elapsed after transplant,^14^ considering that renal dysfunction was the most common reason for sirolimus therapy discontinuation in the study by Bhorade et al.^6^ Therefore, in the first few months after transplant, sirolimus may exacerbate nephrotoxic effects resulting from perioperative stress and high tacrolimus levels, although the study by Sacher et al^14^ found significantly better long-term renal function with sirolimus plus tacrolimus compared with mycophenolate mofetil plus tacrolimus, indicating an eventual renal-sparing effect of sirolimus, consistent with other studies.^17,23^ Finally, the study by Bhorade et al^6^ used induction with basiliximab or daclizumab, whereas our study found that induction therapy was associated with decreased survival among sirolimus-treated patients.

Besides improved survival, we found lower incidence of chronic rejection with sirolimus compared with mycophenolate mofetil and lower death risk after chronic rejection onset, findings that are noteworthy because reports consistently state that patients receiving sirolimus are maintained with significantly lower tacrolimus levels than antimetabolite-receiving patients.^6,14,24^ Although infection incidence was similar between groups, risk of death after infection was significantly lower in the sirolimus group, with a 36% reduction in infection-related deaths during 10 years. This finding suggests that infections occurring in sirolimus-treated patients may be generally less life-threatening than those in antimetabolite-treated patients and is supported by the previously reported finding of reduced cytomegalovirus infections with sirolimus.^6^ Consistent with literature,^8,9,10,11^ our results also suggested a lower incidence of malignant disease with sirolimus, with deaths due to malignant disease occurring 38% less frequently during 10 years.

When considering induction and maintenance therapy together, sirolimus-treated patients who received no antibody induction therapy had the best survival among all induction and maintenance combinations assessed. Median survival associated with sirolimus without induction was almost 3 years longer than survival with induction. One explanation is that in the presence of sirolimus maintenance therapy, the long-term effects of induction^25^ may result in excessive immunosuppression, thereby increasing infections, malignant disease, etc, but not substantially decreasing rejection because sirolimus plus tacrolimus immunosuppression seems effective even without induction therapy. A contrary explanation is that patients receiving induction generally received lower maintenance doses of sirolimus and tacrolimus that were inadequate for preventing rejection. Although our data lack drug doses or levels, this latter explanation may be supported by the fact that the study by Bhorade et al^6^ (in which all patients received induction) reported consistently lower sirolimus and tacrolimus trough levels than in the study by Sacher et al,^14^ and the study by Sacher and colleagues showed better rejection avoidance and survival.

We should clarify that sirolimus use is still appropriate in patients who have received induction therapy because the sirolimus group had at least as good survival as the MMF group among patients who received induction therapy. The key point is that survival appears to be maximized if sirolimus is given without induction therapy, and the benefit of sirolimus apparently exceeds the benefit of any induction drug, even with another maintenance therapy. Importantly, survival within the first year (until the time when sirolimus might be initiated) seems to be unaffected by the absence of induction. The phenomenon of excessive immunosuppression might also explain why the sirolimus plus mycophenolate mofetil plus tacrolimus combination had inferior survival to sirolimus plus tacrolimus alone. This finding suggests that mycophenolate mofetil therapy should be discontinued whenever sirolimus therapy is initiated within a tacrolimus-based regimen except perhaps in special cases (with dosing to avoid overimmunosuppression), such as in patients with severe rejection or especially high rejection risk.

### Practical Considerations

Because sirolimus appears significantly beneficial, efforts should be made to enable patient tolerance and avoid discontinuation of therapy; strategies are described for minimizing and managing the risks of common adverse effects of mammalian target of rapamycin inhibitors.^26^ Unfortunately, we have no data to assess optimal dosages or trough levels. In the study by Sacher et al,^14^ which reported very good survival and encouragingly low incidence of chronic rejection with sirolimus plus tacrolimus, the initial sirolimus dosage at 1 year after transplant was 2 mg/d (adjusted as necessary), and mean prednisone dosages were 7.5 mg/d at 2 years after transplant and 4.5 mg/d at final follow-up (approximately a mean of 7 years after transplant). Mean trough levels at 2 years after transplant were 9.1 ng/mL for sirolimus and 8.6 ng/mL for tacrolimus; at final follow-up, mean trough levels were 7.7 ng/mL for sirolimus and 6.8 ng/mL for tacrolimus.^14^ Similarly high sirolimus trough levels and a low prednisone dosage approximately 1 to 2 years after transplant were reported in a single-arm study using sirolimus plus cyclosporine, with favorable survival and very low incidence of chronic rejection.^16^ Because both studies had relatively young patients (mean ages, 43 and 46 years, respectively), optimal immunosuppressant levels may be lower for older patients. The study by Bhorade et al,^6^ which did not observe a significant benefit of sirolimus, reported consistently lower sirolimus and calcineurin inhibitor levels than the other studies. Overall, these findings suggest that sirolimus therapy, if maintained at adequate trough levels, may be particularly effective at preventing chronic rejection together with a calcineurin inhibitor, although safe drug levels must be carefully evaluated for each patient to avoid overimmunosuppression.

### Limitations and Strengths

This study has several limitations. As a nonrandomized study, it is susceptible to confounding, although our regression analyses were adjusted for an extensive list of potential confounders, and the sirolimus and MMF groups were comparable at the time of transplant in almost all variables examined. Notably, although we controlled for transplant center performance as a likely confounder, the possibility of a general trend remains within individual centers that the physicians who chose to administer sirolimus may have been inherently more (or less) effective in patient management, which could have resulted in an advantage (or disadvantage) for the sirolimus-treated patients. However, given that the sirolimus group consisted of patients from more than 30 centers, a uniform trend of physician-induced confounding in the same direction was unlikely to be consistently prevalent throughout these centers. Some degree of confounding by indication is also plausible, considering that many centers had fewer than 10 patients in the sirolimus group. This suggests that sirolimus was not a typical prophylactic therapy at those centers, so patients at those centers likely started sirolimus therapy within the first year because of some serious complication (besides chronic rejection or malignant disease, which were accounted for) that arose on their initial treatment, and their prognoses may have already been impaired when starting sirolimus treatment. Even at centers that frequently use sirolimus prophylactically, the requirement for delayed initiation poses a potential disadvantage to sirolimus because of possible reluctance to switch patients who are doing very well with their initial antimetabolite to sirolimus, whereas for patients doing poorly, switching to sirolimus may be a favored strategy, thereby also increasing the likelihood that patients with an impaired prognosis would receive sirolimus.

Study strengths include the use of national data containing all US recipients of lung transplants and adjustments for many covariates, including the transplant center, ruling out the explanation that centers that administered sirolimus were inherently higher performing regardless of drug. In addition, we explored multiple approaches to avoid immortal time bias owing to delayed sirolimus therapy initiation, confirming that results were consistent between approaches.

## Conclusions

Using national US lung transplant data, our study suggests that sirolimus is associated with improved survival compared with mycophenolate mofetil within a tacrolimus-based regimen. We found that maximal survival may be achieved if sirolimus is administered without induction therapy, although sirolimus use in patients who have received induction therapy is still associated with favorable survival. Further long-term studies of additional patients undergoing lung transplant are needed to confirm these promising findings associated with sirolimus. In the meantime, to potentially improve patient survival, replacing antimetabolites with sirolimus in the first year if possible, within a tacrolimus-based regimen and ideally without antibody induction therapy, should be strongly considered for all recipients of lung transplant.

## Notes

Supplement. **eTable 1.** Time-Dependent Covariate Analyses **eTable 2.** Survival Comparisons by Induction Therapy Among Sirolimus-Treated Patients **eFigure 1.** Multiple Imputation-Based Classification of Planned Sirolimus Treatment **eFigure 2.** Continued vs Discontinued Sirolimus Treatment 1 Year Later **eFigure 3.** Multiple Imputation-Based Classification of Sirolimus vs MMF With or Without Induction **eFigure 4.** Survival in the First Year, With or Without Induction Therapy
