# Supplementary material for: Survival Associated With Sirolimus Plus Tacrolimus Maintenance Without Induction Therapy Compared With Standard Immunosuppression After Lung Transplant
Source: JAMA Netw Open. 2019 Aug 28;2(8):e1910297. doi: 10.1001/jamanetworkopen.2019.10297 (PMC6716294; doi:10.1001/jamanetworkopen.2019.10297)
Supplement: Supplement. — eTable 1. Time-Dependent Covariate Analyses eTable 2. Survival Comparisons by Induction Therapy Among Sirolimus-Treated Patients eFigure 1. Multiple Imputation-Based Classification of Planned Sirolimus Treatment eFigure 2. Continued vs Discontinued Sirolimus Treatment 1 Year Later eFigure 3. Multiple Imputation-Based Classification of Sirolimus vs MMF With or Without Induction eFigure 4. Survival in the First Year, With or Without Induction Therapy [file jamanetwopen-2-e1910297-s001.pdf]

Supplementary Online Content

Wijesinha M, Hirshon JM, Terrin M, et al. Survival associated with sirolimus plus tacrolimus maintenance without induction therapy compared with standard immunosuppression after lung transplant. *JAMA Netw Open.* 2019;2(8):e1910297. doi:10.1001/jamanetworkopen.2019.10297

eTable 1. Time-Dependent Covariate Analyses
eTable 2. Survival Comparisons by Induction Therapy Among Sirolimus-Treated Patients
eFigure 1. Multiple Imputation-Based Classification of Planned Sirolimus Treatment
eFigure 2. Continued vs Discontinued Sirolimus Treatment 1 Year Later
eFigure 3. Multiple Imputation-Based Classification of Sirolimus vs MMF With or Without Induction
eFigure 4. Survival in the First Year, With or Without Induction Therapy

This supplementary material has been provided by the authors to give readers additional information about their work.

© 2019 Wijesinha M et al. *JAMA Network Open.*

eTable 1. Time-Dependent Covariate Analyses

12-month sirolimus initiation time

	Sir + Tac	MMF + Sir + Tac	MPS + Tac	Aza + Tac	MMF + Tac
**Adjusted HR (95% CI)**	0.66 (0.53–0.82)	1.22 (0.87–1.72)	0.91 (0.76–1.08)	0.93 (0.85–1.01)	1.00 Reference
	p = 0.0002	p = 0.24	p = 0.27	p = 0.07	

3-month sirolimus initiation time

	Sir + Tac	MMF + Sir + Tac	MPS + Tac	Aza + Tac	MMF + Tac
**Adjusted HR (95% CI)**	0.67 (0.55–0.81)	1.09 (0.80–1.47)	0.90 (0.76–1.06)	0.93 (0.86–1.01)	1.00 Reference
	p < 0.0001	p = 0.60	p = 0.21	p = 0.07	

Sir = Sirolimus; MMF = Mycophenolate Mofetil; MPS = Mycophenolate Sodium; Aza = Azathioprine; Tac = Tacrolimus

© 2019 Wijesinha M et al. *JAMA Network Open.*

eTable 2. Survival Comparisons by Induction Therapy Among Sirolimus-Treated Patients

	No Induction	Basiliximab	Daclizumab	Alemtuzumab	Equine ATG	Rabbit ATG
**Adjusted HR**<br><br>**95% CI**<br>**p-value**	1.00<br><br>Reference	2.53<br><br>(1.13–5.65)<br>p = 0.02	1.29<br><br>(0.47–3.57)<br>p = 0.63	2.78<br><br>(0.51–15.02)<br>p = 0.24	2.39<br><br>(0.38–14.82)<br>p = 0.35	5.29<br><br>(0.48–58.07)<br>p = 0.17
**Patients**	67	71	60	15	4	2

ATG = Anti-thymocyte globulin

© 2019 Wijesinha M et al. *JAMA Network Open.*

eFigure 1. Multiple Imputation-Based Classification of Planned Sirolimus Treatment

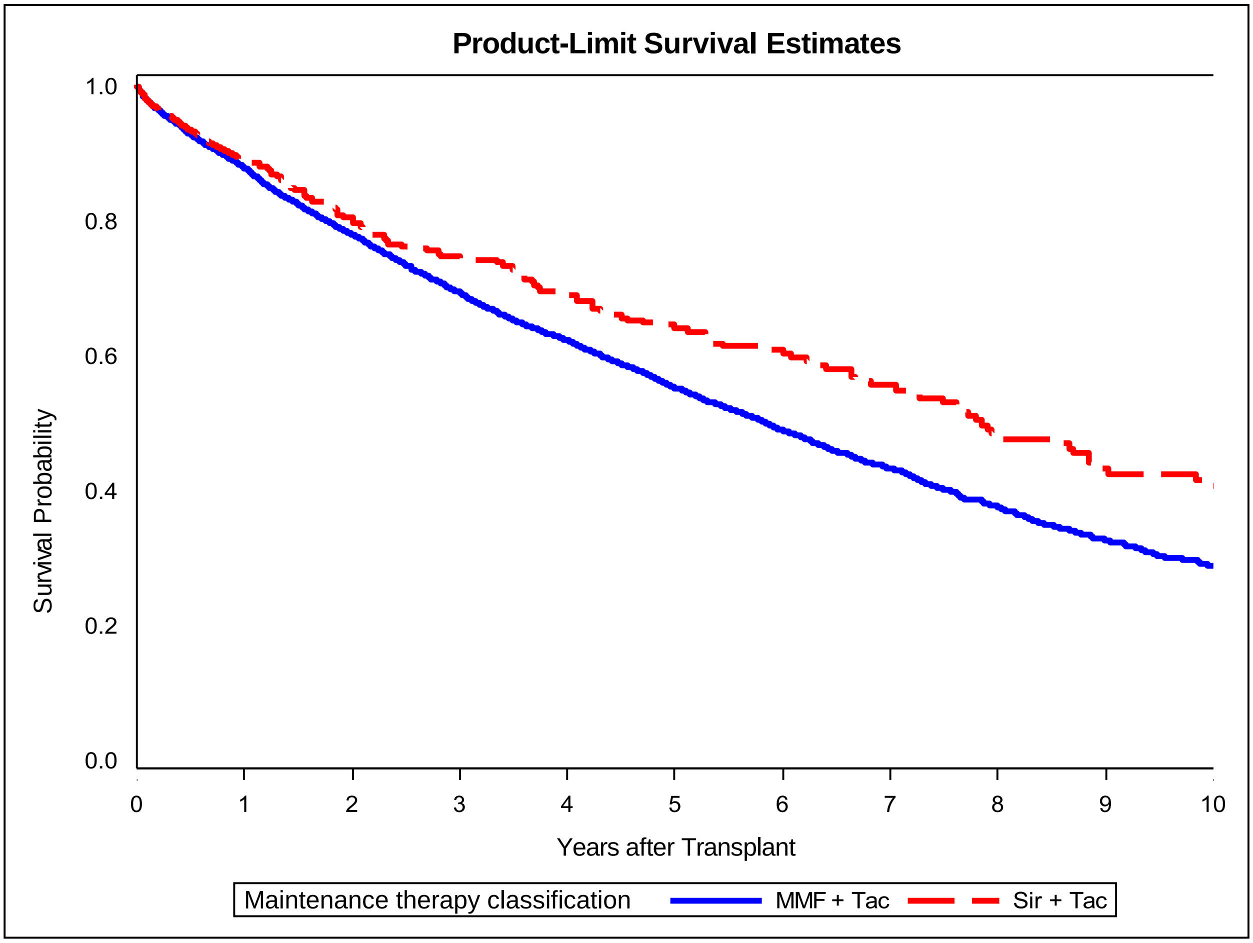

	Sir + Tac	Aza + Tac	MPS + Tac	MMF + Tac
Median Survival (IQR)	7.8 years (2.8–11.9)	6.8 years (2.7–11.4)	6.4 years (2.9–10.0)	5.8 years (2.3–11.1)
10-year survival	41%	34%	26%	29%
Adjusted HR (95% CI)	0.71 (0.58–0.87)	0.93 (0.86–1.01)	0.90 (0.78–1.17)	1.00 Reference
	p = 0.001	p = 0.07	p = 0.21	

Sir = Sirolimus; MMF = Mycophenolate Mofetil; MPS = Mycophenolate Sodium; Aza = Azathioprine;
Tac = Tacrolimus

© 2019 Wijesinha M et al. *JAMA Network Open.*

eFigure 2. Continued vs Discontinued Sirolimus Treatment 1 Year Later

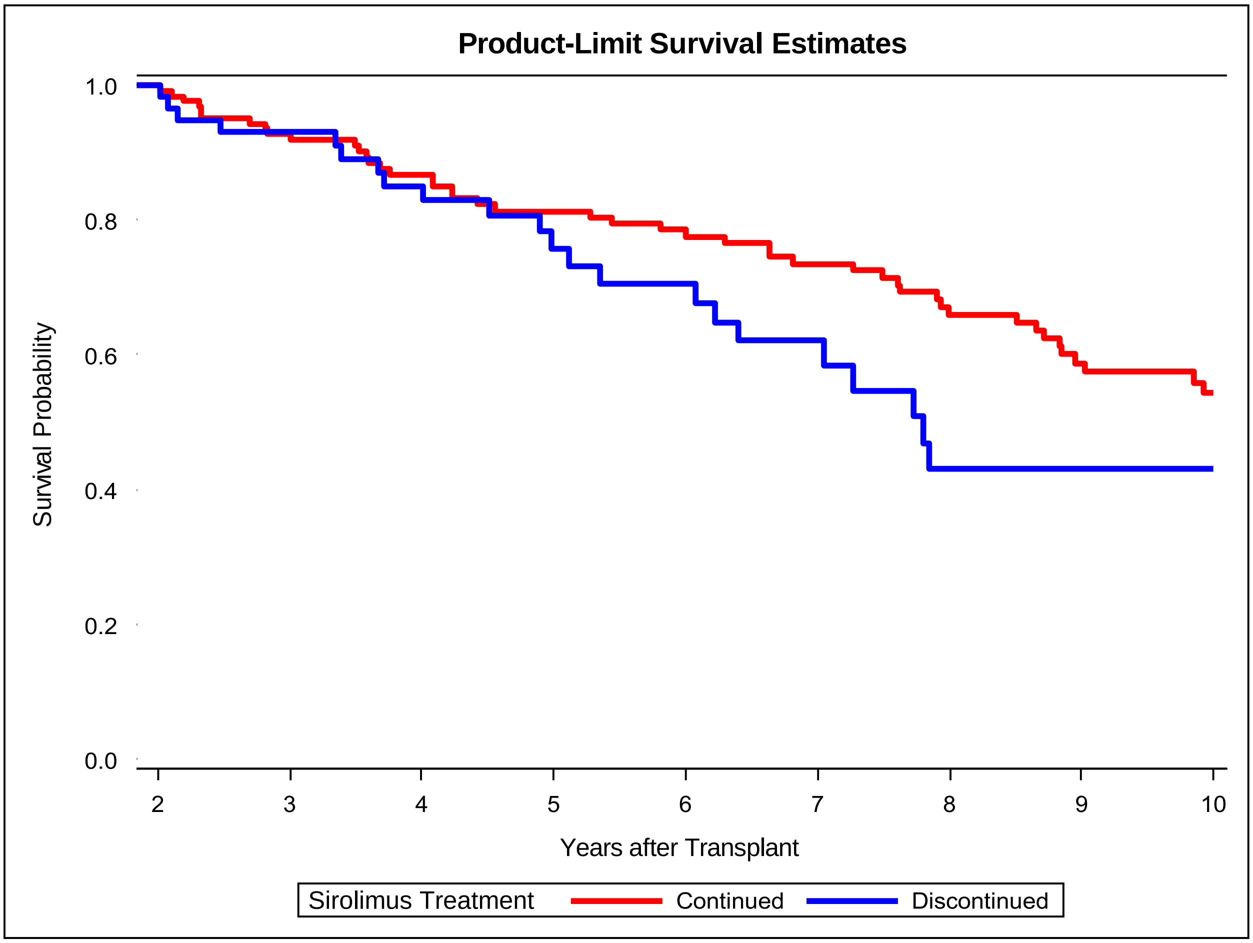

© 2019 Wijesinha M et al. *JAMA Network Open.*

eFigure 3. Multiple Imputation-Based Classifications of Sirolimus vs MMF With or Without Induction

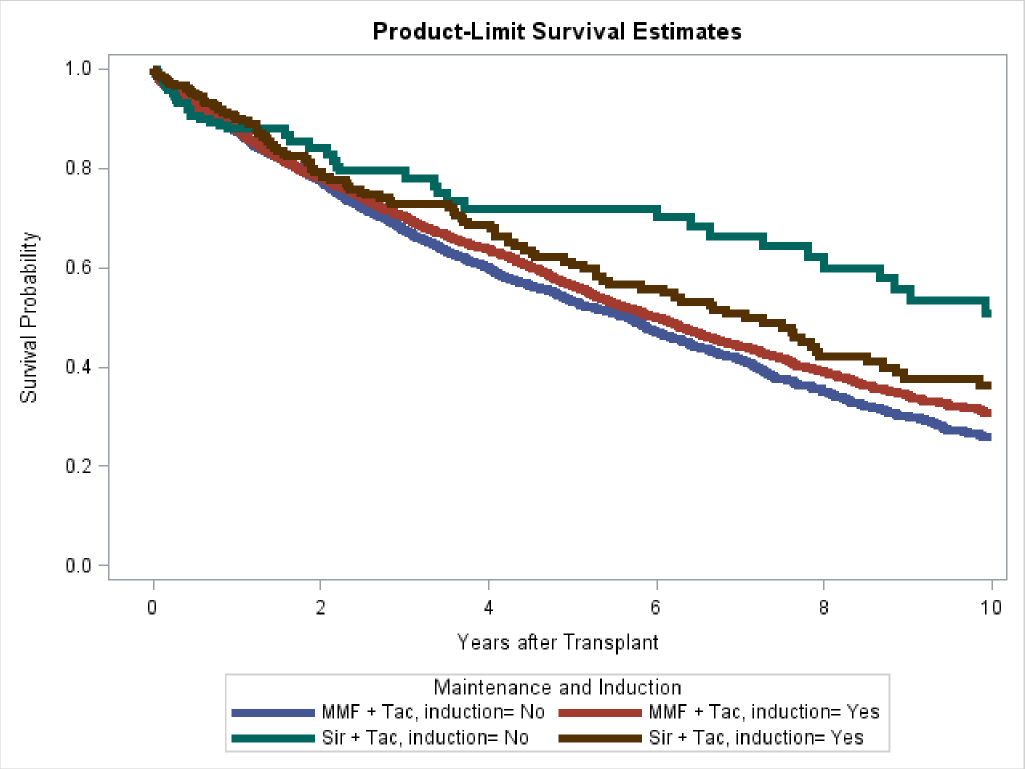

	Maintenance and Induction			
	**Sir + Tac, without induction**	**Sir + Tac, with induction**	**MMF + Tac, without induction**	**MMF + Tac, with induction**
**Median Survival [IQR]**	10.2 years (3.4–12.6)	7.0 years (2.5– -)	5.7 years (2.2–10.5)	6.0 years (2.4–11.5)
**10-year survival**	51%	36%	26%	31%
**Adjusted HR (95% CI)**	**0.52 (0.31-0.77)**	**0.85 (0.67-1.09)**	**1.09 (1.00-1.19)**	1.00 Reference
	p = 0.003	p = 0.19	p = 0.05	

Sir = Sirolimus; MMF = Mycophenolate Mofetil; Tac = Tacrolimus

Induction Therapy includes one of the following:

Alemtuzumab, Antithymocyte Globulin, Basiliximab, Daclizumab

© 2019 Wijesinha M et al. *JAMA Network Open.*

eFigure 4. Survival in the First Year, With or Without Induction Therapy

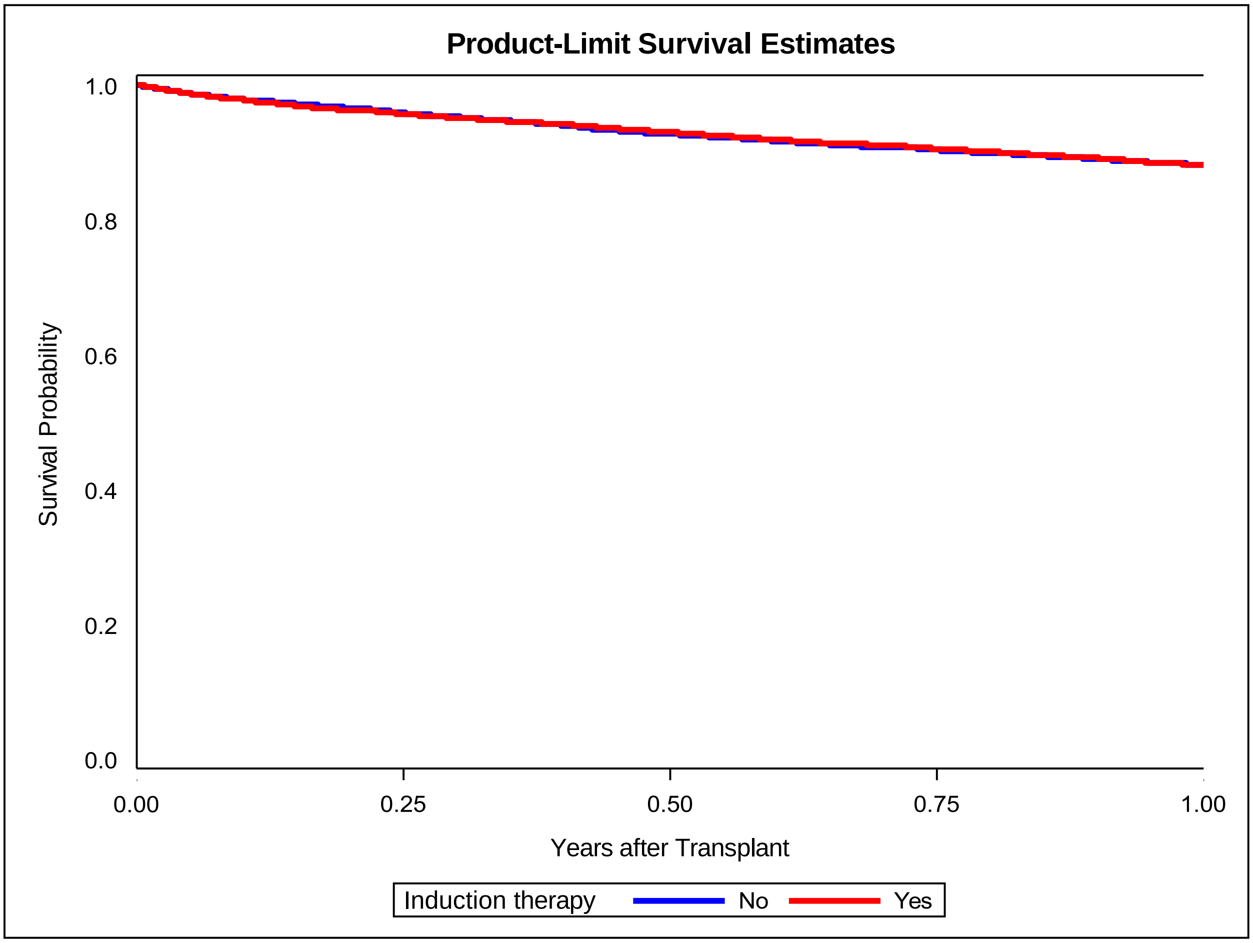

Comparison between induction therapy categories for survival in first year (p=0.24)

Induction Therapy	Patients	1-year survival	Adjusted HR (95% CI)
No Induction	5038	88%	1.00 (Reference)
Basiliximab (Simulect)	4613	88%	1.00 (0.87-1.15)
Daclizumab (Zenapax)	1047	86%	0.98 (0.79-1.22)
Alemtuzumab (Campath / Lemtrada)	699	87%	0.91 (0.66-1.25)
Equine Antithymocyte Globulin [Equine ATG] (Atgam)	599	90%	0.74 (0.53-1.02)
Rabbit Antithymocyte Globulin [Rabbit ATG] (Thymoglobulin)	429	84%	1.30 (0.96-1.76)

© 2019 Wijesinha M et al. *JAMA Network Open.*
